# Advances in the Design of (Nano)Formulations for Delivery of Antisense Oligonucleotides and Small Interfering RNA: Focus on the Central Nervous System

**DOI:** 10.1021/acs.molpharmaceut.0c01238

**Published:** 2021-03-18

**Authors:** Monique C. P. Mendonça, Ayse Kont, Maria Rodriguez Aburto, John F. Cryan, Caitriona M. O’Driscoll

**Affiliations:** † Pharmacodelivery Group, School of Pharmacy, University College Cork, T12 YT20 Cork, Ireland; ‡ APC Microbiome Ireland, University College Cork, T12 YT20 Cork, Ireland; § Department of Anatomy and Neuroscience, University College Cork, T12 XF62 Cork, Ireland

**Keywords:** antisense oligonucleotide, small interfering RNA, blood−brain barrier, systemic delivery, neurological diseases

## Abstract

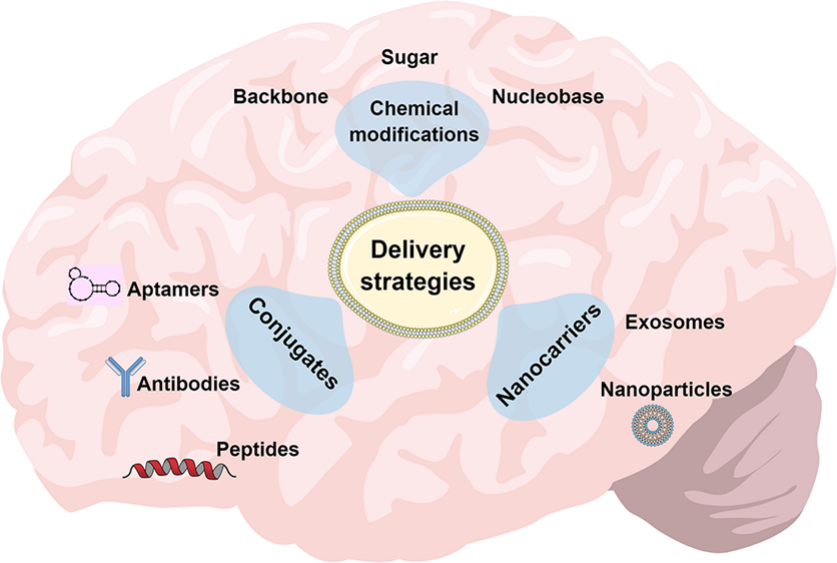

RNA-based therapeutics have emerged as one of the most powerful therapeutic options used for the modulation of gene/protein expression and gene editing with the potential to treat neurodegenerative diseases. However, the delivery of nucleic acids to the central nervous system (CNS), in particular by the systemic route, remains a major hurdle. This review will focus on the strategies for systemic delivery of therapeutic nucleic acids designed to overcome these barriers. Pathways and mechanisms of transport across the blood–brain barrier which could be exploited for delivery are described, focusing in particular on smaller nucleic acids including antisense oligonucleotides (ASOs) and small interfering RNA (siRNA). Approaches used to enhance delivery including chemical modifications, nanocarrier systems, and target selection (cell-specific delivery) are critically analyzed. Learnings achieved from a comparison of the successes and failures reported for CNS delivery of ASOs versus siRNA will help identify opportunities for a wider range of nucleic acids and accelerate the clinical translation of these innovative therapies.

## 1 Introduction

Neurological disorders are the leading cause of disability and the second leading cause of death worldwide.^1^ The incidence of neurological disorders is increasing in tandem with the aging of the population, representing a serious economic burden to society. Therefore, research is urgently needed to develop novel treatments in response to this clinical need.^2^

Gene therapy has emerged as a powerful therapeutic approach for the treatment of neurological diseases. Three different approaches can be used: (i) overexpression of genes, (ii) silencing of the disease-causing gene by RNA interference (RNAi), and (iii) gene editing by the insertion, removal, or replacement of genes in the genome using zinc finger nucleases (ZFNs), transcription activator-like effector nucleases (TALENs), and the recently developed clustered regularly interspaced short palindromic repeats (CRISPR)/associated protein 9 (Cas9) tools.^3,4^

For decades, overexpression of a missing gene product by an exogenous DNA sequence was the only approach for gene therapy. This approach typically utilizes viral vectors as delivery systems, which have been associated with insertional mutagenesis, innate and adaptive immune responses, and toxic effects.^5^ Since the development of RNAi almost two decades ago, this technology has been intensively exploited and many clinical trials have been performed.^6,7^ Antisense oligonucleotides (ASOs) and small interfering RNA (siRNA) are the two most widely studied approaches for silencing gene expression, particularly with respect to the central nervous system (CNS). The main advantages associated with RNA therapeutics are the high specificity to target pathogenic targets, decreasing toxicity associated with off-target effects, and relatively low dose requirement for therapeutic effect.^8−10^ In comparison to RNAi, gene-editing tools exhibit more complex physicochemical and biopharmaceutical properties and are consequently more challenging to deliver especially to the brain.^11^ Thus, this review will focus solely on the gene silencing approach.

Therapeutic targeting of RNA is currently based on two main approaches: single-stranded ASOs and double-stranded RNAi. RNAi process can be mediated by siRNAs, endogenous microRNAs (miRNA), and short hairpin RNA (shRNA). In contrast to siRNA that acts in the cytosol, miRNA and shRNA require transport into the nucleus, an additional barrier to RNAi delivery.^12^ Regarding drug development, siRNA is more suitable for drug use because it does not require genome integration and can be easily synthesized.^13^

ASOs are synthetic single-stranded nucleic acids generally containing 12–30 nucleotides in length (4–10 kDa), designed to bind a target RNA (pre-mRNA, mRNA, noncoding RNA) in a sequence-specific manner via Watson–Crick base-pairing rules. The single-stranded nature of ASOs may result in lower costs and simplify the delivery process when compared to siRNAs.^14^ ASOs regulate RNA modulation either by mRNA cleavage through enzymatic degradation (RNase H) or by an occupancy-only mechanism, sometimes referred to as steric blocking. Furthermore, they can also modulate pre-mRNA splicing, reducing or restoring protein expression.^15−17^

**Figure 1:**
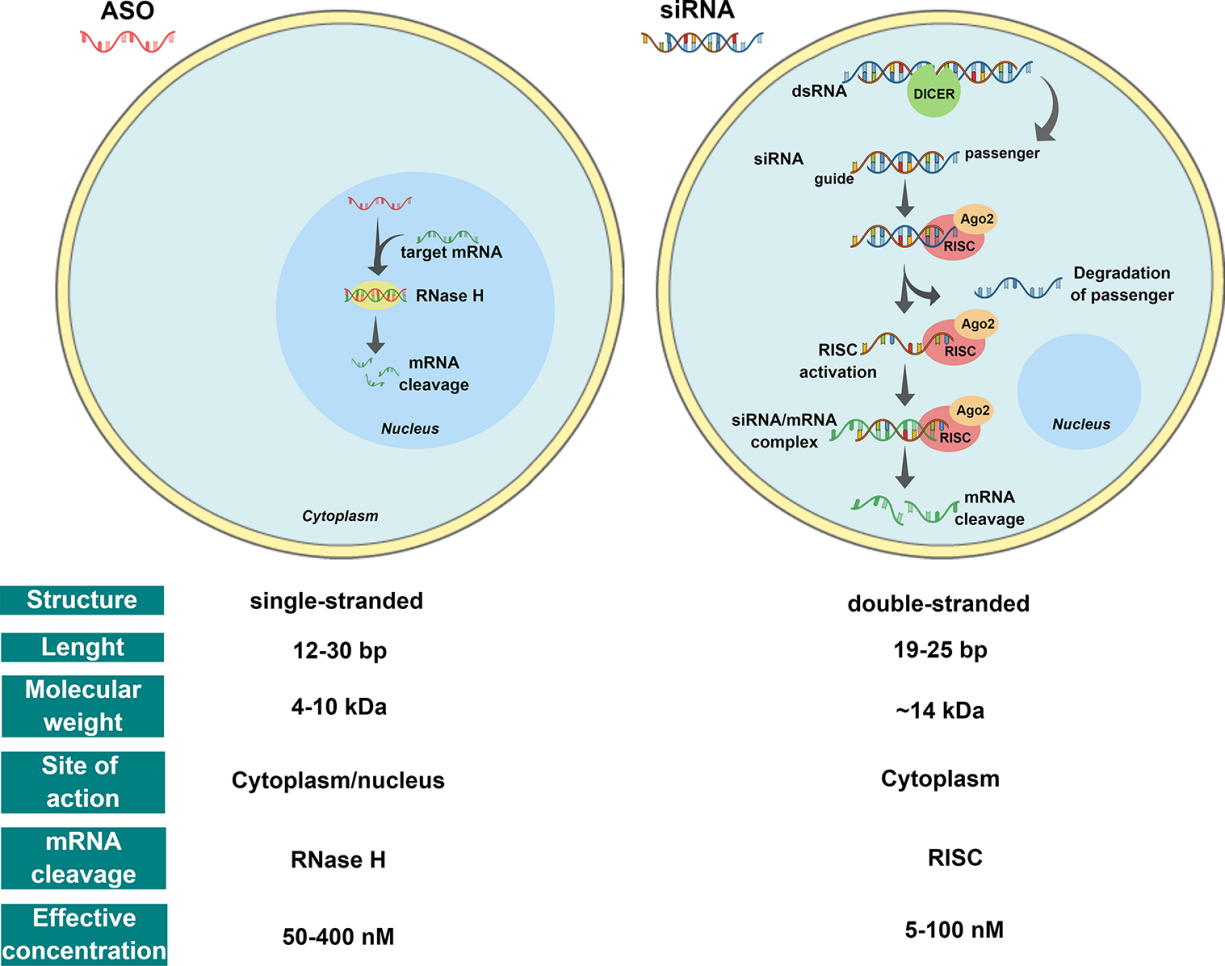
RNAi-based therapeutic main approaches: single-stranded antisense oligonucleotides (ASOs) and double-stranded small interfering RNA (siRNA). ASOs: Once bound to the target mRNA, ASOs can form an RNA–DNA hybrid that becomes a substrate for RNase H, which results in mRNA degradation. siRNA: Double-stranded RNA (dsRNA) is processed by Dicer into siRNA. The guide RNA strand is incorporated into the RNA-induced silencing complex (RISC) and Argonaute 2 (Ago2). Ago2 cleaves the passenger strand, and siRNA/RISC complex then binds the complementary sequence of the target mRNA resulting in the degradation of the target transcript.

RNase H triggering represents the most predominant knockdown mechanism. As illustrated in Figure 1, upon the introduction into cells, ASOs can enter the nucleus and engage with complementary sequences in pre-mRNAs. The formation of a DNA/RNA hybrid results in the recruitment of RNase H1, a ribonuclease that recognizes the DNA/RNA heteroduplex and catalyzes the cleavage of RNA, resulting in reduced mRNA levels.^18,19^

siRNAs are short double-stranded RNA molecules usually containing 19–25 base pairs (∼14 kDa) that regulate gene expression and control a diverse array of biological processes. They consist of two strands: (i) the guide strand (antisense), containing the information for target-gene recognition, and (ii) the passenger strand (sense) required for loading into the RISC (RNA-induced silencing complex). Once in the cytoplasm, the RNAi process starts with the cleavage of long double-stranded RNA into siRNA by the Dicer enzyme (initiation phase). As shown in Figure 1, these small RNAs are then incorporated into the RISC (effector phase). The RISC nuclease Argonaute 2 (Ago2) cleaves siRNA strands and releases the guide strand, resulting in RISC activation. The guide strand anneals with its complementary mRNA leading to its degradation and the silencing of the targeted gene. The activated RISC complex (with antisense strand included) can move on to degrade additional targeted mRNA, allowing transient (3–7 days) gene silencing in rapidly dividing cells and, extending for several weeks, in slowly dividing cells.^19−21^

Although there has been a great progress, the treatment of neurological disorders using nucleic acids remains a challenging issue due to rapid degradation in the circulation, poor cellular uptake, lack of specificity for particular brain cell/tissues, and the complex structure of the brain and physiological barriers, especially the blood–brain barrier (BBB),^8^ which restricts access to the CNS.^22,23^

The BBB is one of the most selective physiological barriers, regulating the transport of molecules, ions, and cells into and out of the brain and protecting the CNS from potentially harmful substances that enter the bloodstream. At the same time, the BBB controls CNS nutrition and homeostasis and maintains the chemical composition of the neuronal milieu required for proper neuronal functioning. The BBB is formed by a monolayer of nonfenestrated endothelial cells sealed by tight junctions (TJ) and adherens junctions (AJ), which together with astrocytes, pericytes, neurons, and the basement membrane constitute the neurovascular unit.^23−25^ The complexity of the BBB restricts CNS drug delivery, thus limiting the treatment of several neurological diseases.

This review will focus on the strategies for the systemic delivery of therapeutic nucleic acids targeting the CNS. Pathways and mechanisms of transport across the BBB which could be exploited for delivery are described, focusing in particular on smaller nucleic acids including ASOs and siRNA. Approaches used to enhance delivery including chemical modifications, nanocarrier systems, and cell-specific delivery are critically analyzed. A comparison of the successes and failures reported for CNS delivery of ASOs versus siRNA highlights learnings, which will help identify future translational opportunities for a wider range of therapeutic nucleic acids.

## 2 Challenges for Systemic Delivery

**Figure 2:**
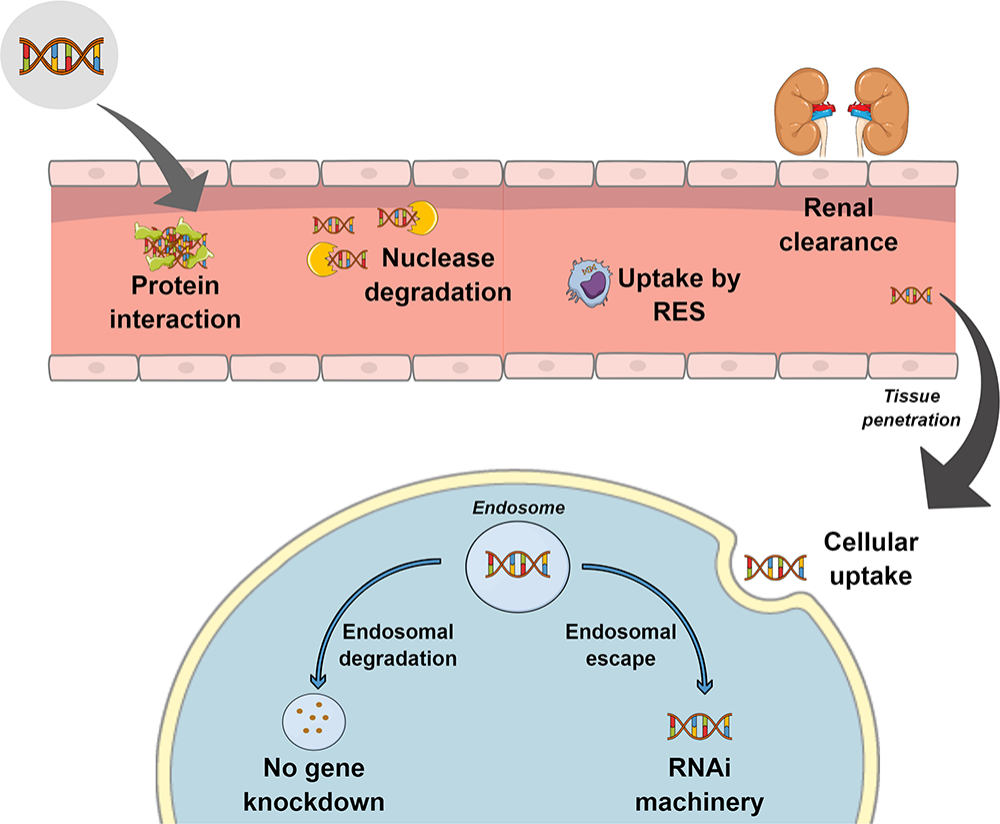
Physiological barriers to systemic delivery of RNA-based therapeutics. After systemic administration, the therapeutic nucleic acid must avoid interaction with bloodstream components, renal excretion, and uptake by phagocytes of the reticuloendothelial system (RES). Once at the targeted tissue, it must be internalized into the cell and escape from the endosome before degradation.

The initial challenges faced by nucleic acids after systemic administration lie in the circulatory system. From the drug delivery point of view, RNA molecules have unfavorable physicochemical properties including a negative charge, high molecular weight and size, and serum instability. Naked RNA molecules are rapidly degraded by nucleases in biological fluids, which results in renal clearance and short circulation time in the blood (<10 min).^12^ Degradation can stimulate the innate immune system, triggering inflammatory and other immune responses and serum protein interaction. This phenomenon, known as opsonisation, causes rapid uptake by the reticuloendothelial system (RES) (Figure 2). After systemic administration, phagocytic cells of RES, specifically the Kupffer cells in the liver and the splenic macrophages, can easily endocytose RNA oligonucleotides, resulting in higher concentrations in these organs following intravenous administration.^13,26^

In addition, the circulating nucleic acids must cross the vascular endothelial barrier to accumulate in the target tissue. The structure and permeability of the capillary endothelia vary across different organs and tissue types, making some tissues more accessible to therapeutics than others. For example, the liver and spleen are composed of fenestrated capillaries and discontinuous basement membranes exhibiting large inter- and intracellular gaps, which allow the easy diffusion of nucleic acids into the tissue interstitium. The CNS capillaries, due to the BBB, exhibit nonfenestrated capillaries with dense intercellular junctional proteins (TJ and AJ) and a continuous basement membrane making extravasation of large molecules into the brain very difficult.^27^

After reaching the target cell, the next challenge is cellular uptake of the nucleic acid therapeutics. The cellular membrane is made of negatively charged phospholipids, and this charge is a barrier for nucleic acid uptake. Moreover, the high molecular weight, large size, and hydrophilic nature of RNA molecules impede membrane permeability. As a result, the major mode of internalization is via endocytosis, whereby the molecules are internalized together with a component of the cell membrane.^12^ Finally, endosomal escape and release into the cytoplasm is a key problem that must be solved to ensure a safe delivery of RNA-based therapeutics and effective gene knockdown.^11^

**Figure 3:**
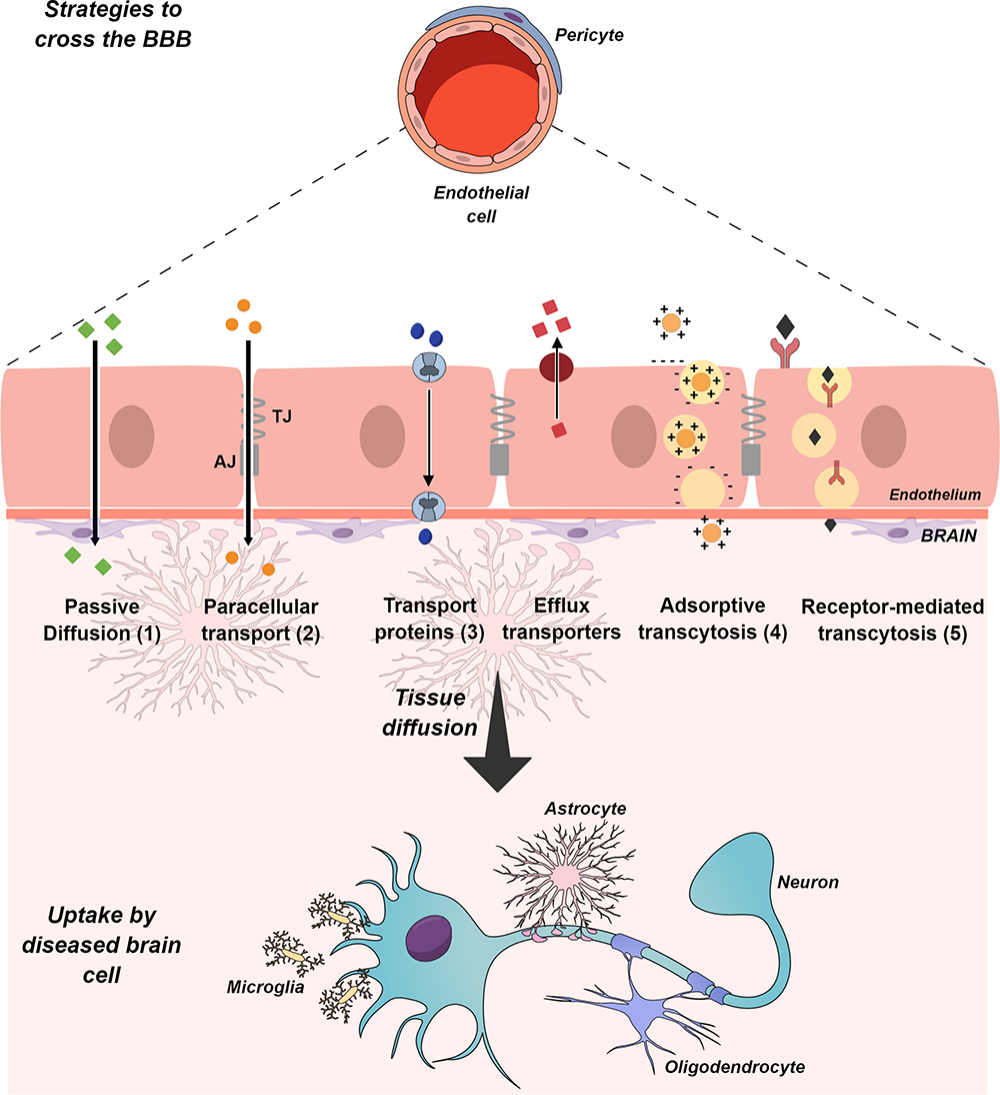
Schematic representation of the blood–brain barrier and the main transport routes for permeation and transport across the endothelium. (1) Small lipid-soluble agents can passively diffuse through the lipid bilayer. (2) Only small water-soluble molecules can diffuse through the intercellular spaces between endothelial cells. (3) The endothelium contains carriers for glucose, amino acids, nucleosides, purine bases, choline, and other substances. (4) Cationic molecules such as albumin and other plasma proteins are taken up by adsorptive-mediated transcytosis, which is consecutive of the endocytosis/exocytosis event. (5) Ligands such as insulin, transferrin, cholesterol-containing particles, and most other protein hormones are taken up by specific receptor-mediated transcytosis. Once across the BBB, the compounds must diffuse toward the disease site and be taken up by the diseased cells. TJ, tight junction; AJ, adherens junction.

In light of the challenges described above, generally, only small lipophilic molecules (<400 Da) freely diffuse across the brain endothelium (Figure 3 (1)). Other small water-soluble molecules can simply cross BBB through the TJ and AJ; however, paracellular transport is generally limited (Figure 3 (2)). Almost all other substances require certain endogenous transport systems to cross the BBB, such as transport proteins (carrier-mediated transport), absorptive-mediated transcytosis, or receptor-mediated transcytosis.^28^

Transport proteins (Figure 3 (3)) enable essential molecules such as glucose, amino acids, monocarboxylic acids, hormones, fatty acids, carbohydrates, nucleotides, inorganic ions, amines, choline, and vitamins to cross the BBB via substrate-specific transporters, e.g., GLUT1 for glucose and LAT1 for some amino acids.^23^ Adsorptive-mediated transcytosis (Figure 3 (4)) is triggered by electrostatic interaction between a positively charged substance and the negatively charged membrane surface of the endothelial cells. Receptor-mediated transcytosis (Figure 3 (5)) enables the transport of small and large molecules including hormones, growth factors, enzymes, and plasma proteins. Endothelial cells have a limited number of receptors on their surface; thus, this route is normally a saturable process.^29,30^

Via receptor-mediated transcytosis, a specific receptor binds to its ligand on the luminal side of the endothelium and carries it to the abluminal side via the formation of endocytic vesicles. These vesicles can be either clathrin- or caveolae-dependent.^31^ Importantly, the different nature of the endocytic vesicles will dictate the intracellular routes followed by the cargo prior to its delivery to the abluminal side (brain). Contrarily to the clathrin-dependent pathway, the caveolae-dependent pathway can bypass lysosomal storage.^32^

The above-described different transport systems across the BBB have been exploited to deliver therapeutic drugs to the brain. Generally, targeting transporters or receptors to access the brain involves creating complexes between the drug of interest and a receptor-targeting item. Such items can be the endogenous receptor/transporter ligands, mimetic ligands, or an antibody targeting the receptor.^32^ Accordingly, transport proteins present in the brain endothelium can be targeted to deliver the drug of interest into the brain. An example for this would be the use of glycosylated nanocarriers that can cross via the GLUT1 receptor.^33^ One clear drawback is that many of these receptors, such as the GLUT receptors, are not exclusively expressed in the brain endothelium, which can lead to nonspecific targeting. Clathrin-dependend transport has also been used for drug delivery into the brain, especially the transferrin receptor. Despite being highly expressed in the brain endothelium, it is also expressed in other tissues throughout the body. Moreover, it is inefficient in delivering the cargo into the brain from the endothelial cytoplasm.^34,35^ Caveolae-dependent transport has an important advantage in terms of intracellular trafficking of the cargo, as it does not involve the lysosomal pathway.^32,36^ In this context low-density lipoprotein receptor is a good candidate to target for drug delivery purposes. To do so, the two main strategies include protein corona-mediated targeting and ligand-based targeting.^35^ Both of these strategies are further detailed below.

Finally, endothelial cells express several ATP-driven drug efflux pumps, such as P-glycoprotein (P-gp), multidrug related protein (MRP), and breast cancer resistance protein (BCRP) transporters, which provide an additional layer of regulation by actively excluding many hydrophobic molecules from the CNS.^29,30^ These efflux transporters constitute an important challenge for drug delivery into brain tissue, as they are known to greatly restrict brain permeability to a wide array of structurally diverse xenobiotics.^37^ Therefore, it is essential that these transporters are considered while assessing drug transport properties in preclinical studies.

In summary, different approaches are being explored in order to overcome the limitations of each of these routes of transport across the BBB. Further investigation will allow optimization of drug delivery into the brain. In the past years, researchers have designed carriers (e.g., nanoparticles (NPs)) to target diseased cells in the brain with high specificity by overcoming the BBB and with reduced toxic side effects.^38^ The main ligands targeting specific receptors on the BBB, as well as cell-specific markers on the diseased brain, are discussed below.

## 3 Design and Development of RNA-Based Therapeutics for Systemic Delivery

Despite the promising potential of RNA-based therapeutics, delivery strategies are required for the successful translation of ASOs and siRNA into the clinic. There are two broad strategies to improve the systemic delivery of RNA-based therapeutics. One is the chemical modification of the ASOs/siRNA structure itself while preserving the molecular nature and activity of the nucleic acid. The other is the incorporation of the nucleic acids into a delivery system. Both strategies aim to enhance the safety and potency of the nucleic acid, resulting in selective and stable systems.^39^

**Figure 4:**
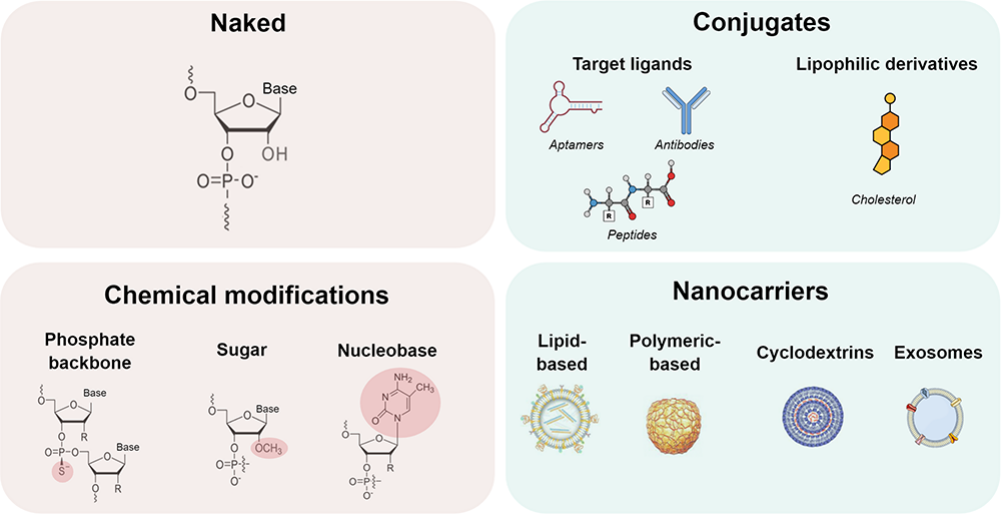
Main strategies for central nervous system delivery of antisense oligonucleotides (ASOs) and small interfering RNA (siRNA). Unformulated/naked and chemical modifications predominate for ASOs, while conjugates and nanocarrier systems have been more widely studied for siRNA.

The main approaches for the delivery of ASOs and siRNA therapeutics are summarized in Figure 4. To date, unformulated/naked and chemical modifications predominate for ASOs, while conjugates and nanocarrier systems have been more widely studied for siRNA.

### 3.1 Chemical Modifications

A variety of chemical modifications have been developed to improve the physical and pharmacological characteristics of RNA-based therapeutics (Table 1), such as ASOs and siRNA. Alterations in the chemical structure of the nucleic acids are focused on either the backbone, the sugar group (ribose), or the nucleobases.^39^

**Table 1:** Common Chemical Modifications Used in RNA-Based Therapeutics^a^

strategy	content	goal
Backbone modifications	Phosphorothioate	Enhance nuclease stability
	Sugar-phosphate	Improve pharmacokinetics
Sugar modifications	2′-Ribose modifications (2′-OMe, 2′-F, 2′-OME, cEt, LNA)	Increase binding affinity to RNA
		Enhance stability against nucleases
		Decrease immune activation
Nucleobase modifications	5-Methylcytosine	Enhance RNA affinity
		Decrease proinflammatory properties
Conjugations	Biomolecules (antibodies, aptamers, peptides)	Modulate protein binding
	Hydrophobic derivatives	Modulate tissue distribution

a Abbreviations: 2′-OMe, 2′-*O*-methyl; 2′-F, 2′-fluoro; 2′-MOE, 2′-*O*-methoxyethyl; cET, 2′,4′-constrained 2′-*O*-ethyl; LNA, locked nucleic acid.

Nucleic acids consist of nucleotides all with a common structure. Nucleotides incorporate ribose linked at the 1′ position to a nucleobase and the 5′ position to a phosphate group. These structures are connected via phosphodiester bonds formed at 3′ and 5′ carbon of the ribose and make up the strands of the nucleic acids. The phosphate linkage and ribose are easy targets for metabolic degradation. Therefore, chemical modifications are preferentially performed at these sites.^40,41^ Regarding their composition, siRNA and ASOs differ in their strand characteristics. siRNA consists of a double-strand and therefore leads to some restriction in the application of modifications due to duplex stability and interaction between siRNA and RISC complex.^42^

#### 3.1.1 Backbone Modifications

The chemistry of the backbone has the largest impact on the pharmacokinetics and pharmacodynamics properties of nucleic acids.^41^ Therefore, the replacement of the natural phosphodiester backbone by the inclusion of a more hydrophobic phosphorothioate (PS) was the first chemical modification implemented on nucleic acids. The PS modification replaces a nonbridging phosphate oxygen atom with a sulfur atom and has been extensively used to improve nuclease stability^43^ of both ASOs^44,45^ and siRNAs.^46^

The PS-modified backbones increase resistance against nucleases in serum and tissues and consequently prolonged circulation time. It promotes protein binding and thus supports interactions with albumin and other blood proteins, thereby retarding renal clearance and ensuring that PS-oligonucleotide can reach the tissues. The PS-modification is fully consistent with the RNase H activity.^41,47−49^ Following intravenous administration of single-stranded PS-modified oligonucleotides, the distribution phase from plasma to tissues ranges from a few minutes to a few hours followed by a prolonged elimination phase that can last for several weeks. In contrast, unmodified oligonucleotides do not bind extensively to plasma proteins and are quickly cleared by the kidneys, accumulating at lower levels in tissues.^15,50^

Similar backbone modifications include the replacement of nonbridging oxygen with boron (boranophosphate), nitrogen (phosphoramidate), or methyl (methylphosphonate).^42,43^ Phosphoramidate and methylphosphonate are preferentially used in ASOs and were not intensively studied in siRNA, whereas boranophosphate is applicable for both nucleic acids.^43^

The replacement of the sugar-phosphate by the introduction of a peptide or morpholino structure leads to the elimination of the charge of the nucleic acid backbone; this modification is mostly used for ASOs. The phosphodiester linkages are replaced with either phosphorodiamidate (morpholino) or polyamide (peptide) linkages.^39,47^

As a result of neutralization, the nucleic acids lose the ability to mobilize RNase H.^47,51,52^ They gain protection against nuclease degradation but operate by translation inhibition through steric interference^52^ and splice modification.^47,51,52^ On the contrary, the neutral character of morpholino- and peptide-modified ASOs impairs cellular uptake due to the decreased probability of interaction with negatively charged cell membranes. To compensate for this drawback, two strategies have been investigated: the formulation into a lipid nanoparticle or the conjugation with a peptide which functions as a targeting ligand, leading to receptor-mediated uptake.^52^

#### 3.1.2 Sugar Modifications

An approach to reduce the immune activation of nucleic acids while further enhancing the nuclease resistance includes substituting the 2′-hydroxy group at the ribose. This approach includes modification with 2′-*O*-methyl (2′-OMe), 2′-fluoro (2′-F), 2′-*O*-methoxyethyl (2′-MOE) groups, and bridged rings (2′,4′-constrained 2′-*O*-ethyl (cET), locked nucleic acid (LNA).^40,41^

All 2′-OH modifications, which can be applied to ASOs, can also be applied to siRNA, though with some restriction due to the mechanism of action of the siRNA. Modifications in siRNA can lead to impaired loading into the RISC complex, which decreases siRNA efficacy. 2′-OMe and 2′-F are the most commonly used modifications in siRNA.^50^ The naturally occurring^40,48^ and therefore nontoxic 2′-OMe modification is only used in alternate bases in siRNA to maintain efficacy and simultaneously gain resistance against recognition by the immune system and also enhanced nuclease resistance.^48^

The 2′-MOE modification is comparatively larger and therefore can only be applied at specific positions in the guide strand of the siRNA because of its negative impact on the silencing activity. Even in ASOs, 2′-MOE is mostly used in the so-called “gapmer” design (see section below).^48^

The bridged 2′,4′-ring of ribose is one of the most complicated 2′-ribose modification involved in ASOs chemistry, especially in the design of gapmer ASOs,^42^ which comprises the binding of cET and LNA.^48^ This modification provides an increased binding affinity to target-mRNA^53^ but is unable to recruit RNase H.^47^ Instead, it operates via alternative splicing or translational inhibition. A new type of oligonucleotide composition was investigated to regain the RNase H recruitment;^52^ this provides the basis for the development of gapmers.^54^ Gapmers contain a central unmodified region of nucleotides, flanked with 2′ modified nucleotides on each side.^47,48^ The unmodified region features RNase H activity,^50^ whereby the flanked regions only improve the binding affinity to target mRNA.^41^ This approach represents high nuclease resistance, low toxicity, and increased hybridization affinities to mRNA.^42,48^

#### 3.1.3 Nucleobase Modifications

Compared to modifications of the backbone and sugar moieties, nucleobase modifications have not been used extensively for the stabilization of RNA-based therapeutics. Modifications to the nucleobases could create modified nucleosides metabolites that may be incorporated into native nucleic acids and interfere with the correct expression and maintenance of genetic material. There is, however, a notable exception: the C-5 methyl substitution on pyrimidine nucleobases (5-methylcytosine, 5-methylcytidine, and 5-methyluridine/ribothymidine).^42,55^ The pyrimidine methylation has the effect of increasing the oligonucleotide melting temperature by ∼0.5 °C per modification and has been commonly incorporated into ASOs (e.g, those under development by Ionis Pharmaceuticals).^56,57^

The nucleobase modification can impact the nucleic acid activity in various ways: nuclease resistance, enhanced sequence selectivity to target mRNA, reduced off-target effects by preventing immune stimulation resulting in decreased proinflammatory characteristics^15,57^ and more efficient gene activity.^58,59^ Interestingly, 5-methyl substitution decreases the activity of siRNA and is therefore not beneficial in this regard.^57^

In summary, as described above, chemical modification is a promising approach to make nucleic acids a successful therapeutic modality to treat diseases including neurological disorders. However, despite the impressive preclinical potential of siRNA for treating brain diseases, most of the candidates, whether approved or in clinical trials, are ASOs. Currently, there are two FDA-approved ASOs: nusinersen (also known as Spinraza, ISIS 396443, ISIS-SMNRx, and ASO-10-27) and eteplirsen (Exondys 51). Nusinersen is indicated to treat spinal muscular atrophy, a hereditary disorder linked to deletion or mutation of the survival motor neuron 1 gene located on chromosome 5q13. It has two chemical modifications, one on its backbone (PS) and another at its sugar units (2′-MOE).^60^ Eteplirsen is an ASO with phosphoroimidate morpholino modification at the backbone against Duchenne muscular dystrophy, a debilitating genetic disease characterized by the lack of functional dystrophin protein, which results in progressive lethal skeletal muscle degeneration.^61^ There are also other candidates in phase 3 clinical trial; Trabedersen is an ASO from Isarna therapeutics design with a simple PS-modification tested against glioblastoma,^61^ and tominersen is a gapmer-RNA modified with 2′-MOE PS developed for the treatment of Huntington disease.^62,63^ Imetelstat, a second glioblastoma product, contains N3′-PS thiophosphorimidate with a covalently linked C16 lipid moiety at the 5′ end.^64^

### 3.2 Nanocarrier Systems

Parallel to the development of chemically modified nucleic acids, researchers have also been working on nucleic acid carrier systems. NPs have a proven track record as efficient carriers for systemic nucleic acid delivery including brain delivery (Table 2). Formulations include lipid-based NPs (LNPs), polymeric NPs, lipid–polymer hybrid NPs, and modified cyclodextrins (CDs). These NPs have demonstrated remarkable properties such as the ability to cross multiple biological barriers and protect target genes against nuclease degradation, improved pharmacokinetic profile by preventing renal excretion and RES clearance, enhanced stability in physiological solutions, and delivery to target specific tissues or cells.^65−68^ Recently, exosomes have also emerged as a new delivery vehicle for siRNA, ASOs, and small molecules to the brain.^69−71^ The characteristics of nanocarrier systems and examples of their formulation and use are reviewed below.

**Table 2:** Selected Examples of Nanoparticle-Based RNA Formulations for *in Vivo* Brain Delivery via Systemic Administration^a^

formulation	nucleic acid	animal model	target	ref
**Lipid-Based NPs**				
Angiopep-2- targeted liposomes	GOLPH3siRNA	U87-GFP-Luciferase-bearing BALB/c mouse	LRP-1	(72)
RVG-9r-targeted liposomes	Ataxin3 siRNAs	C57 BL/6 ataxin-3 [Q69]-transgenic	nACh	(73)
Calcium phosphate lipid NPs	SOD1	Transgenic zebrafish		(74)
	ASO			
**Polymeric NPs**				
GLUT-1 targeted polymeric NPs	MALAT1-ASO	BALB/c mice	Glucose transporter	(75)
PBAE NPs	siRNA	Orthotopic GBM1A mouse model		(76)
**Hybrid NPs**				
Angiopep-2 lipid-PLGA NPs	GOLPH3	Nude U87 xenograft mice	LRP-1	(77)
	siRNA			
**Cyclodextrin**				
Transferrin targeted CD	RRM2	Monkeys	Transferrin receptor	(78)
	siRNA			
**Exosomes**				
RVG-targeted exosomes	BACE1	C57BL/6	nACh	(79)
	siRNA			
T7-peptide targeted exosomes	microRNA-21 ASO	Glioblastoma rat model	Transferrin receptor	(80)

a Abbreviations: CD, cyclodextrin; GOLPH3, Golgi phosphoprotein 3; LRP-1, low density lipoprotein receptor-related protein 1; nACh, nicotinic acetylcholine receptor; NPs, nanoparticles; PBAE, poly(β-aminoester); RRM2, ribonucleotide reductase subunit M2; RVG, rabies virus glycoprotein; SOD, superoxide dismutase I.

#### 3.2.1 Lipid-Based NPs

Over the past decades, the delivery approach that is both most extensively used and most clinically advanced is to complex oligonucleotides with cationic lipids, thus forming LNPs, also called lipoplexes.^16,50,81^ In 2018, the first-ever RNAi therapeutic, Onpattro (Patisiran), was approved by FDA and launched by Alnylam. Onpattro is formulated as a LNPs to delivery siRNA targeting transthyretin (TTR) into hepatocytes for the treatment of hereditary TTR-mediated amyloidosis in adults.^82^ This drug represents the dawn of the RNA nanomedicine era, and it further accelerated the development of nucleic acid-loaded LNPs for various therapies. Recently, BioNTech/Pfizer and Moderna encapsulated their mRNA vaccines against COVID-19 using LNPs.^83^

Lipid-based NPs include liposomes, solid lipid NPs, and nanostructured lipid carriers. These nanocarrier systems are composed of cationic (ionizable) lipids that bind DNA or RNA molecules, neutral helper lipids that increase transfection efficiency, and a nucleic acid vector encoding for the target gene.^84^ Sometimes an additional targeting ligand is also incorporated to enable selective delivery to targeted cells after systemic administration. Generally, the basic structure of the cationic lipid employed for gene delivery consists of three domains: a hydrophilic headgroup (monocation or polycation, linear or heterocyclic) attached, via a linker bond, to a hydrophobic tail group (cholesterol or aliphatic).^85^ The positively charged headgroup not only is responsible for the nucleic acid complexation but also affects NPs characteristics such as the surface charge. A large number of cationic headgroup structures have been investigated for application in gene delivery; selected examples are shown in Table 3.^84^

**Table 3:** Cationic Lipids That Have Been Used in Lipid-Based NPs^a^

amino lipid	optimized ionizable lipid	lipidoid
Monovalent cationic: DOTAP; DOTMA; DMRIE	Dlin-DMA	C12-200
Monovalent ionizable: DODAP; DODMA	Dlin-KC2-DMA	98N12-5
Multivalent ionizable: DOGS	Dlin-MC3-DMA	
Cholesterol derivatives: DC-Chol; GL67		

a Abbreviations: DC-Chol, 3β-(*N*-(*N*′,*N*′-dimethylaminoethane)carbamoyl)cholesterol; DLinDMA, 1,2-dilinoleyloxy-3-dimethylaminopropane; DMRIE, *N*-(2-hydroxyethyl)-*N,N*-dimethyl-2,3-bis(tetradecyloxy)-1-propanaminium bromide; DODAP, 1,2-dioleoyl-3-dimethylammoniumpropane; DODMA, 1,2-dioleyloxy-3-dimethylaminopropane; DOGS, *N*,*N*-dioctadecylamidoglycylspermine; DOTAP, *N*-[1-(2,3-dioleoyloxy)propyl]-*N*,*N*,*N*-trimethylammonium methyl sulfate; DOTMA, *N*-[1-(2,3-dioleyloxy)propyl])-*N*,*N*,*N*-trimethylammonium chloride; GL-67, N4-spermine cholesterylcarbamate

Although permanently charged cationic lipids have proven useful for *in vitro* transfection purposes, their utility *in vivo* is limited due to reduced transfection efficiency and cellular toxicity. The toxicity is normally associated with higher charge ratios between the cationic lipids and the nucleic acids as well as the dose administered.^86^ To overcome this issue, ionizable cationic lipids such as DODAP were developed with apparent p*K*_a_ values between 6 and 7.^87^ This p*K*_a_ ensures an efficient encapsulation of nucleic acid polymers at acid pH, a near neutral or mildly charged surface in the circulation at physiological pH, and a high positive surface charge at the acid environment of the endosome, which destabilized the NPs to release their RNA cargo.^88^

The rational design of the linker and the tail group is another strategy to enhance the efficacy of the formulation. The linker affects not only the global p*K*_a_ of ionizable lipids but also the size, flexibility for charge presentation, and biodegradability of the delivery system. The lipid properties of the tail group such as the degree of saturation, chain length, and substitution also affect the transfection efficiency.^84^

Several groups have encapsulated nucleic acids into LNPs for brain targeted gene delivery using the aforementioned ionizable cationic lipids.^73,89−91^ Cohen et al. developed LNPs composed of the ionizable cationic lipid DLin-MC3-DMA, helper lipids distearoylphosphatidylcholine (DSPC), and cholesterol, using dimyristoyl glycerol (DMG), PEG (DMG-PEG), and distearoylphosphatidylethanolamine (DSPE)-PEG amine as linkers. The formulation was further functionalized with hyaluronan to deliver PLK1-siRNA to glioma cells. The authors observed a robust silencing of 80% in PLK-1 expression and prolonged survival (+60%) of a U87 xenograft mouse model.^89^ Conceição and coauthors use DODAP, cholesterol, and DSPC to formulate liposomes. The liposomes were further functionalized with the brain-targeting rabies virus glycoprotein (RVG)29–nona-arginine, and conjugated to DSPE-PEG-maleimide. Promising results were observed in two transgenic mouse models of Machado–Joseph disease (MJD), also called spinocerebellar ataxia type 3 (SCA3), upon intravenous administration of siRNA targeting mutant ataxin-3 mRNA. The efficient silence of mutant ataxin-3 reduced the neuropathology and motor behavior deficits of both mice strains.^73^

A further liposome-mediated delivery has been developed (DCL64) composed of dipalmitoylphosphatidylcholine, cholesterol, and poloxamer L64. Intravenous administration of DCL64 formulation resulted in the interaction with low-density lipoprotein receptors (LDLr and LRP-1) and subsequent accumulation of the oligonucleotides in Purkinje cells of mouse cerebellum.^92^ In another study, cationic liposomes with angiopep-2, a specific ligand of LRP-1, were used to deliver Golgi phosphoprotein 3 (GOLPH3)-siRNA for glioma treatment. Using an U87-GFP-luciferase-bearing BALB/c mouse model, the authors demonstrated that the liposomes delivered GOLPH3-siRNA specifically to glioma and effectively inhibited glioma growth.^72^

#### 3.2.2 Polymeric Nanoparticles

Polymeric NPs provide another widely used strategy for nucleic acid delivery. Although they have not progressed clinically to the same degree as LNPs, polymeric NPs have shown desirable features such as biological safety (low immunogenicity, absence of mutagenesis), chemical versatility, facile synthesis, and low production costs.^50,93,94^

At an early stage, natural polymers such as polysaccharides (chitosan, CDs, alginate) and proteins (gelatin, albumin) were investigated as sustained gene delivery vectors; however, they exhibited low transfection efficiency. Therefore, in an effort to increase transfection efficacy many synthetic polymers have been developed including poly(ethylenimine) (PEI), poly(lactic acid) (PLA), poly(l-glutamic acid) (PLGA), poly(l-lysine) (PLL), poly-ε-caprolactone (PCL), poly(β-aminoester) (PBAE), poly(2-(dimethylamino)ethyl methacrylate) (PDMAEMA), and the dendrimer poly(amidoamine) (PAMAM). Synthetic polymers have been used alone or in combination with natural polymers in the preparation of NPs or incorporated into sustained-release systems such as hydrogels, nanospheres, microspheres, and scaffolds.^94−96^ Regardless, PEGylation, functionalization with targeting ligands, or modification by introducing histidine residues in their backbones is still necessary to improve the transfection efficiency and circulation times of polymeric NPs.^97^

The most commonly explored compounds for brain gene delivery include the polymers PEI, PBAE, and PLGA and the dendrimer PAMAM.^96,98,99^ However, without surface modification with target ligands, polymeric NPs have a limited capacity to cross the BBB in sufficient amounts for therapeutic application. In a recent study, GLUT-1 targeted polymeric NPs (glycosyl-PEG-PLL modified with 3-mercaptopropyl amidine and 2-thiolaneimine) were designed for the delivery of ASOs across the BBB. The nanocarrier demonstrated efficient brain accumulation 1 h after intravenous administration and exhibits significant knockdown of a target long noncoding RNA in various mouse brain regions, including the cerebral cortex and hippocampus.^75^

#### 3.2.3 Lipid–Polymer Hybrid Nanoparticles

**Figure 5:**
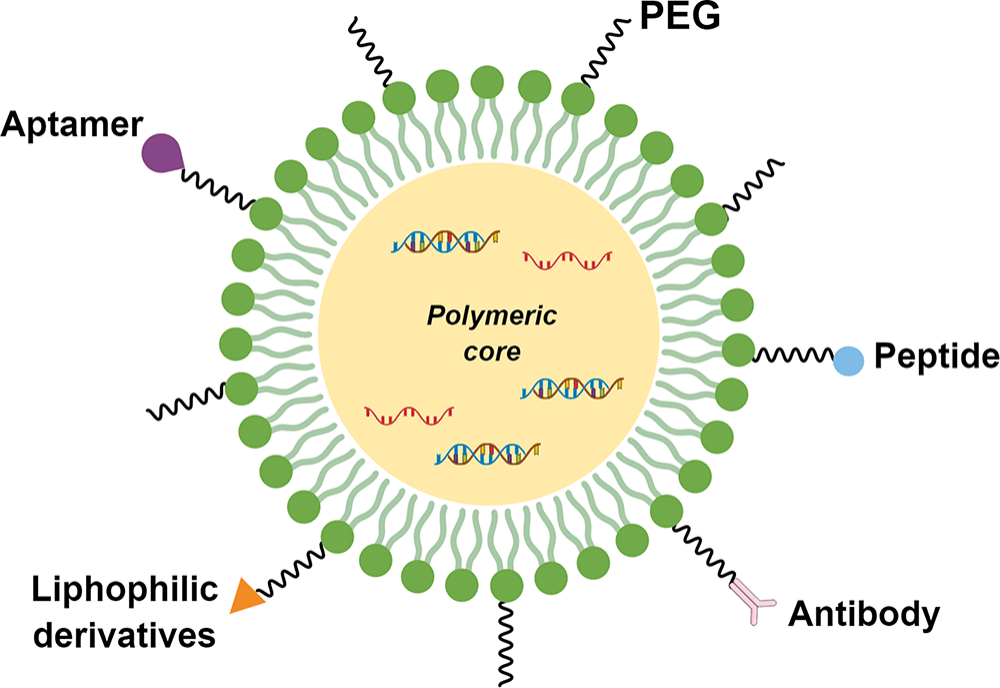
Schematic representation of a lipid–polymer hybrid nanoparticles (NPs). The NPs comprise a polymeric core containing a payload (siRNA or ASOs) surrounded by a lipid shell. Note that an additional outer PEG layer can be added and conjugated with targeting moieties such as aptamers, peptides, antibodies, and lipophilic derivatives (e.g., cholesterol). PEG, polyethylene glycol.

Lipid–polymer hybrid NPs for gene delivery (lipopolyplexes) combine the desirable features of both lipids and polymeric NPs with high *in vivo* transfection efficiencies, improved colloidal stability, and reduced cytotoxicity.^97^ As shown in Figure 5, this type of nanostructure generally has a polymeric core containing the therapeutic agents to be delivered and a lipid shell that may either be a monolayer or bilayer. In some cases, an additional outer PEG layer and target ligands are further coated onto the lipid surface.^100^

An efficient delivery system based on DOTAP, PLGA, DSPE-PEG_2000_, and Angiopep-2 was developed to co-deliver gefitinib and GOLPH3-siRNA across the BBB. The authors demonstrated that Angiopep-2 improved siRNA delivery to the brain tumor, downregulated GOLPH3 and EGFR expression after intravenous administration, and increased the median survival of the animals by 30% compared to nontreated controls.^77^

#### 3.2.4 Cyclodextrins

**Figure 6:**
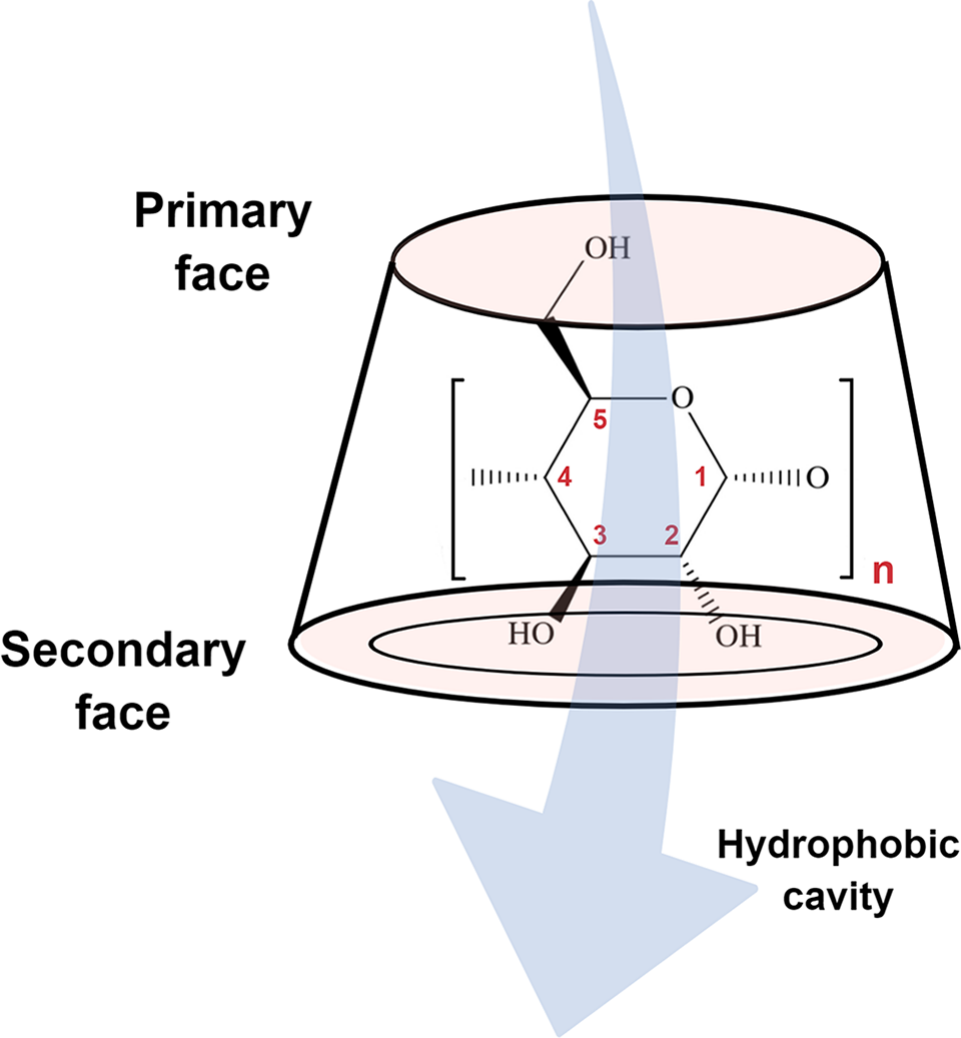
Schematic illustration of the 3D structure of cyclodextrin (CD). The CD comprises glucose units linked by α-1,4-glycosidic bonds and has a hydrophobic central cavity and a hydrophilic outer surface.

CDs are a family of naturally occurring cyclic oligosaccharides obtained through bacterial digestion of starch, which are composed of glucose units linked by α-1,4-glycosidic bonds. The most common forms are α, β-, and γ-CDs, which consist of six, seven, and eight d-glucopyranose units (*n* = 6, 7 or 8), respectively.^101^ CDs have a truncated cone-shaped appearance with upper (narrow, primary) and lower (wide, secondary) rims. The core is relatively hydrophobic due to the presence of CH groups and glycosidic oxygens, whereas the hydrophilicity at the cavity entrances (rims) is attributed to primary and secondary hydroxyl functional groups (−OH) (Figure 6).^102^ Such unique structure results in an “inner–outer” amphiphilic characteristic enabling the CDs to encapsulate organic and inorganic molecules via host–guest interaction.^101^ Due to these features and excellent biocompatibility and low toxicity, CDs have been profusely exploited by the pharmaceutical industry to improve the solubility of hydrophobic compounds and/or to improve stability, bioavailability, and delivery of hydrophilic as well as lipophilic drugs through biological membranes^101,103,104^

In the past decade, attention has been focused on the potential application of modified CD as gene delivery systems,^105−107^ especially for treating brain diseases.^108−111^ Modified amphiphilic β-cyclodextrins were used to deliver Huntingtin (HTT) targeted siRNAs to *in vitro* and *in vivo* models of Huntington disease. The formulation was stable in cerebrospinal fluid with limited toxicity. Sustained knockdown effects were observed associated with the alleviation of motor deficits.^108^

Besides its potential as a carrier, CDs can also act as therapeutic agents to treat neurodegenerative disorders exhibiting impaired cholesterol metabolism (e.g., Niemann–Pick type C (NPC), Alzheimer’s, Huntington’s, and Parkinson’s diseases). CDs can directly interact with the BBB endothelial cells and modify cell membrane composition due to their ability of lipid extraction that directly influences cholesterol trafficking and homeostasis.^104^ Two clinical trials are currently underway to evaluate the safety and pharmacokinetic and pharmacodynamic assessments of systemic iv administration of hydroxypropyl-β-cyclodextrin to NPC patients.^112^

#### 3.2.5 Exosomes

Exosomes have recently emerged as a novel delivery system for biological therapeutics including siRNAs, ASOs, antibodies, and small molecules, especially those targeted to the brain tissue that require passage through the BBB.^69−71^ Exosomes are extracellular nanovesicles (40–120 nm) produced by almost every cell type, including B cells, T cells, dendritic cells, macrophages, neurons, glial cells, astrocytes, stem cells, and most tumor cell lines. They are generated mainly when multivesicular bodies fuse with the plasma membrane and release their content (exosomes) to the extracellular milieu. After exocytosis, exosomes are taken up by the recipient cells and release their cargo (e.g., nucleic acids, enzymes, peptides, lipids), which can mediate many physiological and pathological processes.^113,114^

Owing to desirable properties such as size, lipid membrane bilayer structure, and functional properties, exosome-based delivery has multiple advantages over other delivery systems. For instance, exosomes are stable in the bloodstream, enabling prolonged circulation. They are capable of carrying soluble drugs due to their hydrophilic core. Because exosomes are nanosized and are isolated from specific cells, they have a high capacity for overcoming various biological barriers, possess a natural targeting capacity, and thus have fewer off-target effects^71,113^

Pioneering work on exosome-mediated systemic CNS delivery of siRNA was initiated by Alvarez-Erviti and co-workers in 2011. Murine self-derived dendritic exosomes targeted with the neuron-specific RVG peptide and the exosomal membrane protein Lamp2b delivered GAPDH siRNA and BACE1 siRNA to neurons, microglia, oligodendrocytes in the mouse brain after intravenous administration. The exposure to RVG-decorated exosomes resulted in strong mRNA and protein knockdown of BACE-1 (∼60%), a therapeutic target in Alzheimer’s disease.^79^ Similarly, a T7 peptide-decorated exosome (T7-exo) was produced by incorporation T7, a transferrin receptor-binding peptide, into the exosome membrane as a fusion protein of T7 and Lamp2b. The T7-exo was evaluated as a carrier for brain-targeted delivery of antisense miRNA oligonucleotides against miR-21 (AMO-21), using RVG exosomes/AMO-21 as a control. Both brain-targeting ligands RVG and T7 increased the targeted trafficking of exosomes to the brain and reduced miR-21 levels in the glioblastoma by ∼80% and ∼60%, respectively, compared to the control group.^80^

### 3.3 Nucleic Acid Conjugates

Although significant progress has been made with chemical modifications and nanocarriers systems, brain-specific targeting is essential for an improved therapeutic effect of RNA-based therapeutics. To overcome the hurdles of selective CNS delivery of RNA-based technologies, reliable transport ligands have been linked to nucleic acids whether presented in naked form, as a chemical conjugate, or in association with a nanocarrier. Covalent conjugation of specific molecules to nucleic acids is a promising therapeutic approach to improve cellular uptake as well as pharmacokinetic properties.^40,115^ However, conjugates can be applied to relatively small nucleic acid molecules such as ASOs and siRNA, but it is difficult to apply to large macromolecules such as mRNA, plasmid DNA, and CRISPR/Cas9. This method includes forming conjugates with (1) biomolecules capable of specifically binding receptors to the cell membrane such as folate, antibodies, aptamers, some peptides, and carbohydrates; (2) molecules capable of cell penetration by natural transport mechanisms (e.g., cholesterol and vitamins), or (3) molecules capable of interacting nonspecifically with the cell membrane such as positively charged compounds.^116^

These ligands target specific receptors on the BBB, as well as cell-specific markers on the diseased brain. The most widely studied ligands are transferrin, insulin, and low-density lipoprotein (LDL) receptors. Additional ligands that may have enhanced BBB specificity include aptamers, antibodies, peptides, and lipophilic derivatives.^116−118^ The conjugation of siRNA (naked form) or NPs containing siRNA with ligands for targeted brain delivery will be discussed in detail below

#### 3.3.1 Aptamers

Nucleic acid aptamers are short, single-stranded DNA or RNA oligonucleotides that assume unique tridimensional structures capable of specific molecular recognition of their cognate target. Generated through a process named systematic evolution of ligands by exponential enrichment (SELEX), aptamers demonstrate high affinity and specificity similar to the way that monoclonal antibodies bind to antigens.^119^ Since aptamers can be synthetized against any target, they can potentially be used for the specific delivery to any organ, tissue, or cell where the desired target would be expressed.^120^ Moreover, they have some crucial advantages, such as low immunogenicity and toxicity, prolonged stability, and low production variability.^121^

Aptamers have been identified as highly promising agents for brain-targeted therapy due to the ability of some aptamer conjugates to cross the BBB.^122−124^ A bifunctional aptamer targeting the transferrin receptor (TfR) and the epithelial cell adhesion molecule (EpCAM), a cell surface marker overexpressed on several solid tumors has been developed. Since transferrin receptors are highly expressed on the surface of the BBB, TfR-EpCAM aptamer can bind to transferrin receptors on the surface of endothelial cells to be transported across the BBB and therefore deliver the payload to EpCAM-positive cells. In this study, the resulting bispecific TfR-EpCAM aptamer showed enhanced binding affinity and was able to effectively transcytose through an *in vitro* BBB model and also in healthy NOD/SCID mice following a single intravenous injection (40 nmol/kg).^123^

In recent years, aptamers have been transformed into multifunctional agents for the selective delivery of siRNAs, microRNA, small hairpin RNAs, and ASOs,^121^ the so-called aptamer-chimeras. Aptamer-siRNA chimeras may offer dual functions in which the aptamer inhibits a receptor function while the RNAi internalizes into the cell to target a specific mRNA. Using a coculture model of human endothelial cells, astrocytes, and pericytes, it has been shown that both GL21.T and Gint4.T aptamers, either as single molecules or conjugated to microRNA-137 or anti-microRNA-10b, can cross the BBB. The RNA aptamers, GL21.T and Gint4.T, were able to bind with high affinity and inhibit the intracellular signaling of tyrosine kinase receptors (RTKs) Axl and the platelet-derived growth factor receptor β (PDGFRβ), respectively. These receptors are frequently highly expressed in glioblastoma stem-like cells and are associated with neovascularization.^124^ A Gint4.T aptamer has successfully delivered STAT3 siRNA (Gint4.T-STAT3) to glioblastoma cells resulting in the silencing of STAT3 in PDGFRβ^+^ glioblastoma cells, thereby reducing cell viability and migration. Importantly, Gint4.T-STAT3 reduced tumor growth and angiogenesis *in vivo* in a subcutaneous xenograft mouse model after repeated systemic injections.^125^

#### 3.3.2 Antibodies

Monoclonal antibodies (mAbs), which act as a molecular “Trojan horse”, have been adopted to deliver large molecules across the BBB. The receptor specific mAb penetrates the BBB via transcytosis mediated by specific receptors on endothelial cells, commonly the insulin and transferrin receptors.^126^ For example, OX26 (anti-rat TfR monoclonal antibody), R17-217 and 8D3 (both anti-mouse TfR monoclonal antibody), and 83-14 (anti-human insulin receptor) have all been investigated.^127,128^

The ability of a mAb against the human insulin receptor (HIRMab) combined with avidin–biotin technology successfully delivered siRNA across the BBB in an *in vivo* brain cancer model. Intravenous administration of the antibody-siRNA conjugate led to an efficient (69–81%) suppression in luciferase gene expression.^129^ Following the same principle, antibodies against antigens expressed on glioblastoma stem cells (CD44 and EphA2) were conjugated to chemically modified ASOs against renal cell carcinoma (DRR), also called FAM107A, a genetic driver of glioblastoma invasion. The therapeutic conjugate was successfully internalized and reduced DRR/FAM107A expression in patient-derived glioblastoma stem cells.^130^

Despite decades of development, the use of antibodies remains limited due to the need for sophisticated production and purification equipment leading to high costs. A new generation of optimized antibodies including antibody fragments or diabodies are now emerging to tackle this limitation, but complex manufacturing processes remain a challenge.^120^

#### 3.3.3 Cell-Penetrating Peptides

Cell-penetrating peptides (CPPs) are short peptidic sequences, generally with 5–30 amino acids, which facilitate drug or CPPs/cargo complexes to translocate across the cellular membrane. CPPs are classified into two categories: (1) based on the origin of peptides (synthetic, chimeric, protein-derived peptides) and (2) based on the physicochemical properties (cationic, amphipathic, and hydrophobic).^131,132^ These short peptides are ligands for specific receptors that facilitate cell internalization by endocytosis and destabilization of endosomal compartments.^133^

Several BBB shuttle peptides with increasing efficiency and versatility have been reported including Angiopep-2, apolipoprotein (Apo) B, ApoE, peptide-22, THR, Leptin30, MiniAp-4, RVG29, RVG-9R, GSH, G23, TAT (47–57), and octa-arginine (R8).^134,135^ The short peptide RVG, which is known to specifically bind to acetylcholine receptors in neuronal cells, was the first CPP used in the transport of oligonucleotides into healthy mouse brains. Intravenous administration of RVG-9R siRNA complexes to wild-type Balb/C mice induced a significant reduction in both mRNA and protein levels of Cu–Zn superoxide dismutase (SOD1) in the brain.^136^ Examples of other studies designed to deliver nucleic acids into the brain using RVG constructs have been discussed in detail above.^73,79,80^

As a further demonstration of CPPs delivery potential, Angiopep-2 modified PLGA NPs have successfully co-delivered doxorubicin and epidermal growth factor receptor (EGFR) siRNA for glioma therapy. This delivery system was capable of penetrating the BBB *in vivo*, resulting in extended survival of the glioma-bearing mice and cell apoptosis in the glioma tissue.^137^

#### 3.3.4 Lipophilic Derivatives

Lipophilic molecules are often conjugated to oligonucleotides for delivery purposes. To date, lipophilic conjugates have been used for the delivery of both single- and double-stranded RNAs.^138^ The concept behind conjugation with these molecules involves naturally occurring cell membrane transport mechanisms. More specifically, oligonucleotides modified with cholesterol are recognized by high- and low-density lipoproteins (HDL and LDL, respectively) and internalized via cholesterol binding receptors.^133^ Additionally, the addition of these lipid moieties to oligonucleotides increases the lipophilicity of the nucleic acids and enhances their permeability across the cell membrane.^116^

Conjugation of siRNA with cholesterol, fatty acids, and vitamins (with or without a phosphocholine polar headgroup) has been shown to modulate siRNA tissue distribution and silencing activity *in vivo*.^139,140^ In general, lipid-conjugated siRNAs primarily accumulate in clearance tissues (liver, kidney, and spleen). Higher lipophilic siRNAs preferentially bind LDL and distribute to the liver, whereas less lipophilic compounds bind to HDL in serum and accumulate in kidneys. No perfect correlation between compound accumulation and efficacy has been observed.^139,140^

Regarding the brain, the degree of distribution is strongly and inversely correlated with the hydrophobicity.^141^ Although highly hydrophobic cholesterol-conjugated modified siRNAs (Chol-hsiRNAs) presented limited spread from the site of injection after intrastriatal injection, both docosahexaenoic acid (DHA)-conjugated, hydrophobic siRNA (DHA-hsiRNA) and phosphocholine-containing DHA-hsiRNA conjugate (PC-DHA-hsiRNA) diffused to other brain regions further away from the striatum and induced 70–80% silencing at both mRNA and protein levels.^142,143^

#### 3.3.5 Protein Corona (Endogenous Ligands)

Upon systemic administration, NPs encounter serum components, such as proteins, in the biological fluids resulting in the formation of a protein corona on the surface. Although protein corona formation has been associated with undesirable effects, recent studies suggest that corona-mediated targeting by controlling the function of target plasma proteins on nanosurface may provide a more specific drug delivery.^32,144−146^

With regard to the brain targeting, apolipoproteins play a key role. They are involved in the intercellular transport of insoluble lipids to various cell types, which are taken up via specific apolipoprotein-recognizing receptors (e.g., LDL receptor and scavenger receptor class B type I (SR-B1)) expressed in several tissues and in the brain.^147^ For instance, Zhang et al. obtained a successful corona-mediated brain-targeting using a liposomal system loaded with doxorubicin and functionalized with a peptide derived from the amyloid β-protein (Aβ_1–42_). When exposed to biological milieu, this peptide specifically interacts with the lipid-binding domain of the brain targeting apolipoproteins (i.e, ApoA1, ApoE, and ApoJ), resulting in the exposure of their receptor-binding domain. The reengineered liposomes demonstrated high brain-targeting capacity and improved anticancer effects compared to nontarget plain liposomes.^145^ By use of a different approach, lipid NPs have been prefunctionalized with ApoE4 before systemic administration. This strategy increased NPs translocation into brain parenchyma and exhibited a 3-fold improvement in brain accumulation compared to undecorated NPs.^146^

Despite these promising results, there is no available data concerning the exploitation of corona proteins for systemic delivery of nucleic acid. However, lessons learned from these studies could offer further insights into formulations designed for targeted delivery of nucleic acids to the brain.

## 4 Clinical Progress

Currently, there are a large number of preclinical studies focusing on the delivery of siRNA-based therapeutics into the brain. Although these drugs have not yet reached clinical trials, the successful delivery of siRNA to non-CNS tumor tissue using nanoparticle-based delivery systems following systemic administration has been demonstrated in multiple trials, providing proof-of-concept for RNAi-based therapeutics in humans.^148^ Two nonviral siRNA drugs, namely, Onpattro (patisiran) and Givlaari (Ggivosiran), discussed in section 3.2.1, have already reached the market, and there are some other siRNA-based drugs in the pipeline for approval in the coming years.^149^

Almost two decades after the discovery of RNAi therapeutics, several challenges have limited the usefulness of siRNA in clinical trials for brain delivery.^8^ To date, the main obstacles are delivery, limited diffusion/distribution, and durability (Figures 2 and 3). After injection directly into the brain, siRNA shows effective silencing for only a short period and remains regionally restricted to cells near the injection site. Similarly, after injection into the spinal cord, siRNA does not penetrate broadly into the brain parenchyma and requires several weeklong continuous perfusions to achieve efficacy. On the other hand, ASOs is effective for several weeks after a single intrathecal dose.^150^ These and other challenges, such as clinical trial design and commercial considerations, have limited the usefulness of siRNA therapeutics and will require further optimization to produce successful drugs for brain disease therapy. With the increase in research addressing these challenges and with the recent emergence of siRNA-based drugs in the market, the hope is that the application of siRNA-based therapeutics for the treatment of neurological diseases will also be exploited in clinical settings in the near future.

Although systemic administration is more acceptable for patients compared to direct brain injection, this is not a common route of administration since nucleic acids do not cross the BBB after systemic administration. To simplify the delivery problem, drugs have been designed for administration directly into the brain and/or spinal cord. However, there are concerns regarding technical complications and the risks from highly invasive neurosurgery for patients. The complications are associated with the inaccurate insertion of the catheter, management of the device (including refills of the drugs), the possibility of an allergic reaction or rejection, infections, side effects, and tissue damage with each local administration.^151^ Thus, safe and systemic delivery is the key focus in the development of novel nucleic acid delivery systems targeting the CNS.

The recent approval by the U.S. Food and Drug Administration (FDA) of two ASO-mediated therapies for the treatment of Duchenne muscular dystrophy (eteplirsen, Exondys 51) and spinal muscular atrophy (nusinersen, Spinraza) has opened a new era in nucleic acid-based therapeutics for neurodegenerative diseases.^10,152^ To date, more than 100 ASOs are in preclinical development.^153^Table 4 summarizes some ASO-based therapeutics that have advanced from the bench to the clinical stages. The application of siRNA to neurodegenerative diseases is not as advanced as ASO technology.

**Table 4:** ASO-Based Therapeutics for the Treatment of Brain Disease That Are FDA-Approved or Currently in Clinical Trials^a^

drug	disease	route	status	sponsor	NCT number
ASOs					
IONIS MAPTRx	Alzheimer disease	Intrathecal	Phase 1	Ionis Pharmaceuticals	NCT03186989
BIIB094	Parkinson disease	Intrathecal	Phase 1	Ionis Pharmaceuticals	NCT03976349
WVE-120101	Huntington disease	Intrathecal	Phase 1/2	Wave Life Sciences	NCT03225833
WVE-120102	Huntington disease	Intrathecal	Phase 1/2	Wave Life Sciences	NCT03225846
Imetelstat	Glioblastoma brainstem tumors	Intravenous	Phase 2, terminated	Geron	NCT01836549
Tominersen^b^	Huntington disease	Intrathecal	Phase 3, recruiting	Roche	NCT03842969
					NCT03761849
Trabedersen	Glioblastoma	Intratumoral	Phase 3, terminated	Isarna therapeutics	NCT00761280
Eteplirsen (Exondys 51)	DMD	Intravenous	FDA approved	Sarepta Therapeutics	
Nusinersen (Spinraza)	Spinal muscular atrophy	Intrathecal	FDA approved	Biogen	

a Abbreviations: ASOs, antisense oligonucleotides; DMD, Duchenne muscular dystrophy. NCT: ClinicalTrials.gov identifier number. Data were collected from ClinicalTrials.gov and ref (153).

b Tominersen (previously known as IONIS-HTTRx and RG6042).

## 5 Future Directions

Targeted gene modification via gene-editing tools (ZFNs, TALENs, and CRISPR/Cas9) has been emerging as a new therapeutic option for the treatment of neurodegenerative diseases.^154,155^ However, there are still relevant drawbacks that need to be overcome before their clinical implementation. The major challenge is increasing the specificity and efficiency by decreasing the off-targets side effects. Furthermore, these components are more challenging to deliver, especially to the brain, due to the complexity and high molecular weight.^11,156^ It is anticipated that research in gene editing will continue and advance significantly in the coming years.

A lesson learned from the research performed to date is that delivery tools do not necessarily adapt to all applications. The gene therapy approaches developed thus far have their own advantages and limitations, and therefore, choosing the best tool largely depends on the situation and clinical need.

## 6 Conclusions

This review highlighted the chemical modifications and nanocarriers systems that are currently under investigation for ASOs and siRNA delivery to the brain. There are many opportunities to optimize the formulation design to allow for systemic delivery. A combination strategy for delivery including chemical modification in tandem with a smart delivery system designed to achieve stability in the circulation, permeability across the BBB, diffusion to the diseased site, and specific uptake by the diseased cells may help the translation of these therapeutics for neurodegenerative diseases.

In summary, the past two decades have seen an exponential increase in RNA-based therapies. Several clinical trials have been approved, are ongoing, or completed, with successful launches worldwide; however, RNAi has not yet achieved its full therapeutic potential. It is expected that new RNA-based medicines capaable of reaching nonliver or nontumor tissues following systemic administration will be produced in the coming years.
